# [Retracted] Silencing of long non-coding RNA SNHG15 suppresses proliferation, migration and invasion of pancreatic cancer cells by regulating the microRNA-345-5p/RAB27B axis

**DOI:** 10.3892/etm.2025.12919

**Published:** 2025-07-07

**Authors:** Pengfei Jiang, Youmin Yin, Yan Wu, Zhaoli Sun

Exp Ther Med 22:1273, 2021; DOI: 10.3892/etm.2021.10708

Following the publication of this paper, it was drawn to the Editor’s attention by a concerned reader that certain of the Transwell invasion assay data shown in Fig. 2D (namely, the ‘si-NC’ data for the PANC-1 cell line) on p. 6 were strikingly similar to data that had already appeared in different form in another article written by different authors at different research institutes, which was already under consideration for publication at the journal *Technology in Cancer Research & Treatment* at the time when this paper was submitted to *Experimental and Therapeutic Medicine*.

In view of the fact that the abovementioned data had already apparently been submitted for publication before the receipt of this article at *Experimental and Therapeutic Medicine*, the Editor has decided that this paper should be retracted from the Journal. The authors were asked for an explanation to account for these concerns, but the Editorial Office did not receive a reply. The Editor apologizes to the readership for any inconvenience caused.

